# Development of Parkinsonism symptoms immediately after severe head injury

**DOI:** 10.17712/nsj.2017.4.20170240

**Published:** 2017-10

**Authors:** Khandaker Abu Talha, Mohammad Sulaiman, Deepak Joshi, Sameh Hamed

**Affiliations:** *From the Department of Neurosurgery, King Faisal Hospital, Taif, Kingdom of Saudi Arabia*

## Abstract

This is case report of a young adult male who had developed Parkinsonism symptoms shortly after severe head injury due to accidental fall. This 45-year-old man was diagnosed as multiple hemorrhagic contusions, sub arachnoid hemorrhage and linear skull fracture. After 4 weeks of fall, he developed Parkinsonian symptoms which were relieved by anti-parkinson’s disease medication. After doing thorough journal review only few reports were found regarding short term onset of Parkinson’s disease after severe head injury, though most of the literatures evident of the similar symptoms after more than years after the head trauma.

Among all the neurodegenerative disorders the Parkinson’s disease (PD) is second commonest. Rigidity, resting tremor, postural/ balance impairment and bradykinesia are the chief clinical manifestations of PD. Apart from the motor symptoms the patient could also have cognitive impairment, depression and olfaction dysfunction.^1^ This relationship between PD and head injury got more attention when Muhammad Ali diagnosed as PD at the age of 42 year.^1^ After the diagnosis of Muhammad Ali lots of studies were carried out to determine the risk factor. Few of them were reported head injury as a potential risk factor while few others didn’t find any relationship.^2^ The odds ratio of the risk factor of head injury for PD ranged from 1.4 – 11.7.^3^ Degeneration of the dopaminergic neurons in the pars compacta of the substantia niagra is the main pathological findings of Parkinson’s disease. This leads to loss of dopamine in striatum. Clinical features become prominent when 80% of the dopeminergic receptors are depleted.^4^ Parkinson’s diesease is also characterized by mitochondrial dysfunction, defective handling of proteins, oxidative stress and inflammation, and presence of alpha(α)-synuclein Lewy body in the surviving dopaminergic neurons. Alpha-Synuclein is a protein that has been reported as one of the important neurodegenerative biomarkers in both sporadic and familial PD.^4^

The onset of developing PD after head injury is more than years in most of the cases. There are only few reports of developing PD immediately after head injury. In this report the middle age young adult male patient who suffered from severe head injury, developed parkinsonian features immediately after his head trauma. The aim and objective of this case report is to create a hypothesis for the future research on the same topic. It will also provide an opportunity for a future case series on head injury patients developing Parkinsonism.

## Case Report

This 45-year-old previously healthy right-handed Saudi male was brought to the Emergency Room of the hospital with generalized convulsion. He had alleged history of head trauma due to accidental fall. On the time of arrival his Glasgow Coma Scale (GCS) was 8/15. He did not have any motor weakness. His both pupils were equal and reacting to light and did not have any lateralizing sign. He was intubated immediately and was connected with mechanical ventilator. The gentleman was shifted to Intensive Care Unit and his convulsion was controlled by intravenous anti-convulsant medicine. His vitals were stable all through. Computerized Tomography (CT) scan brain was reported as right frontal contusion, right parieto-temporal small contusion, right fronto temporal sub arachnoid hemorrhage and right temporal linear fracture. The CT brain angiogram was normal.

**Figure 1:**
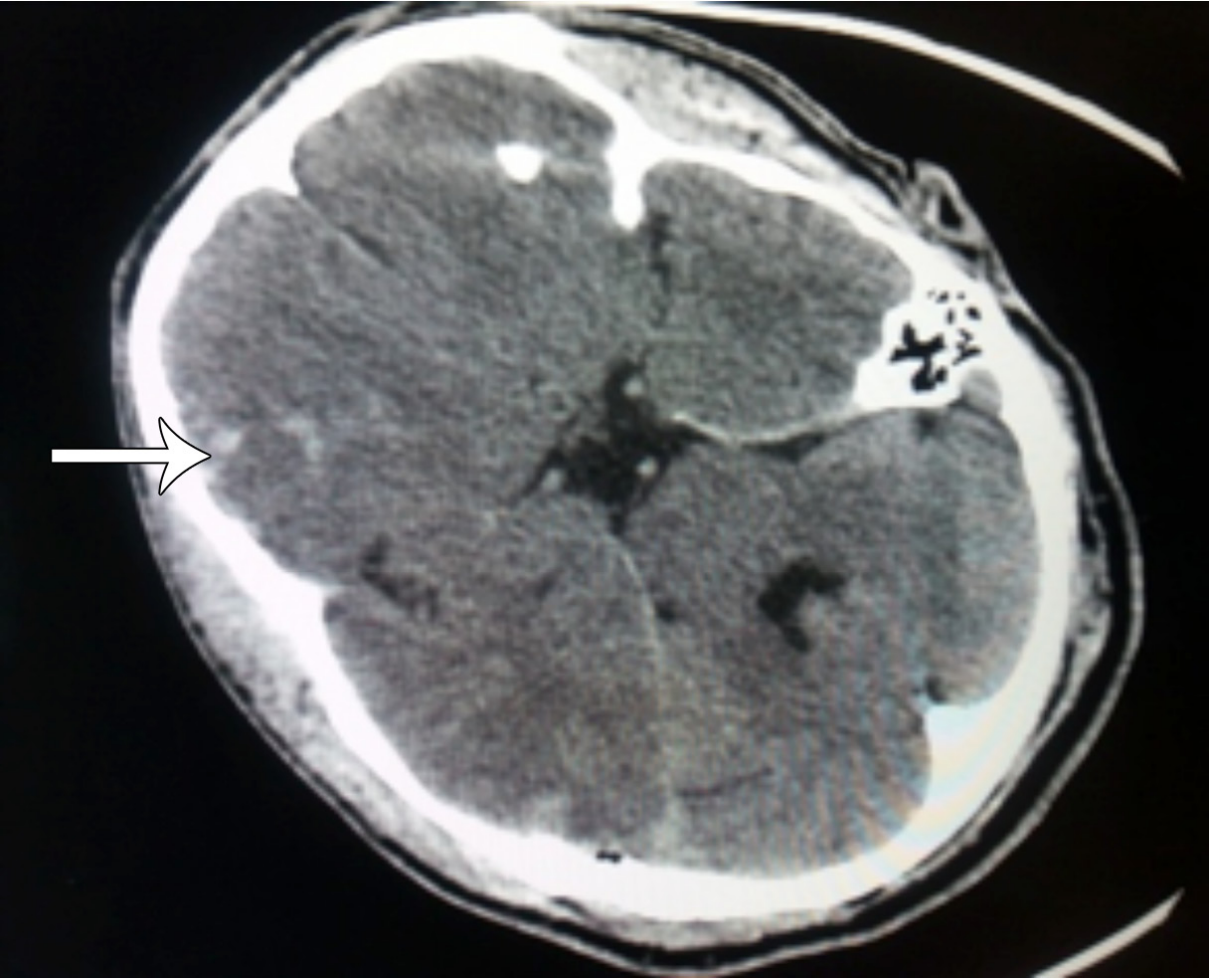
Computed tomography scan of brain at the time of admission (arrow).

**Figure 2:**
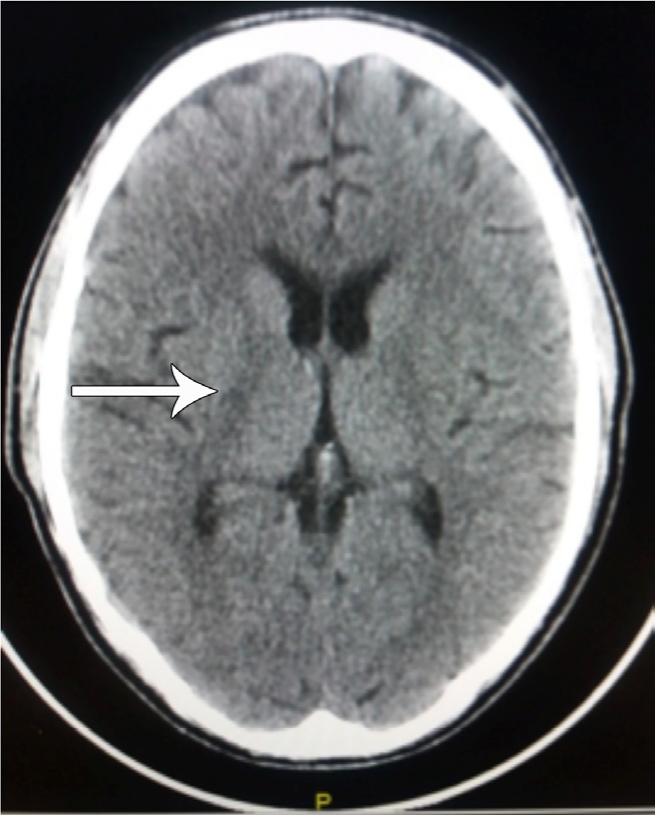
Computed tomography scan of brain at the time of developing PD symptoms (arrow).

Within first 5 days, several attempts were made to wean him off ventilator but all the attempts were failed due to his increased heart rate and repeated convulsion. Neurology consultation was sought and other 2 anti-convulsant medicines were added to control the convulsion. Electro Encephalography was performed which was reported as unremarkable. Tracheostomy was carried out on seventh day of his admission and eventually he was disconnected from ventilator machine after 5 days of tracheostomy.

His tracheostomy tube was removed on 21 nd of admission. His GCS was 14/15 after removal of tracheostomy tube. As his vitals were stable and he was convulsion free, so he was transferred to the cabin with 24 hours watcher attendance. At this point, he was receiving only phenytoin as convulsion prophylaxis. He was able to take oral diet and started normal verbal communication.

Nearly 4 weeks after admission, he developed abnormal rolling movement in his right thumb and index finger. He also developed limb spasticity and rigidity. Neurologist examined the patient and revealed akinesia and positive glabellar tap. He was diagnosed as Parkinson’s disease. Anti-Parkinson’s disease medicine (Sinemet 12.5/50 mg tablet twice a day) was started and gradually he became sign and symptom free. At the time of diagnosing PD his biochemical parameters including phenytoin level were normal. He was not taking any anti-dopaminergic drug. The early development of the parkinsonian symptoms within 4 weeks after severe head injury gives us an opportunity to present the rare scenario.

## Discussion

Jafari et al^5^ have performed their systemic review and meta-analysis on 34 articles.^5^ The Odd Ratio of the study was 1.57. They concluded as head injury was significantly associated with Parkinson’s Disease.

Gardner et al^6^ published their study on different mechanism of head injury and its relationship with Parkinson’s disease. Also they have measured the severity of head injury in relation to PD. Developing PD was significantly more in traumatic head injury group. When the result was compared between falls versus non-fall group it was found there was no significant difference. Patients of moderate or severe head injury patients were more prone to develop PD in comparison to mild head injury patients. Average time of developing PD after head injury was 5-7 years. The study of Gardener^6^ was performed on more than 165 thousand patients. The PD patients were categorized according to age also. When head trauma was due fall then it was more likely to develop PD.^6^ In their study average time of developing PD after head injury was 3.3 years. The mean age of developing post head injury PD was 76 years. The study showed a female (59%) predominance in gender and white (68%) in race. According to this report the patient was a young adult male. The etiology of head injury was fall but the symptoms developed within 4 weeks of head injury. In this report the patient had head injury due to fall. The head injury was severe in classification and the symptoms developed very early in comparison the average time mentioned by Gardner.

Marras et al^7^ have review 165 reports on the same topic.^7^ They found 5 of them had low bias level. Sample size was more than 30 in all studies. Among these 5 studies only 1 study concluded to have association between PD and mild traumatic head injury.

Doder et al^8^ have published a case report on a 36 year old man who has developed PD 4 weeks after head trauma. He was unconscious for more than 24 hours after having the injury. He had skull fracture in CT scan. His PD feature was predominant in right side. He was unresponsive to levodopa therapy. In this report the onset of PD was in almost same time in comparison the report of Doder M et al.^8^ The features were also prominent in the same side.

McKee and Robinson^9^ had their study on 196 subjects. They found no increased risk of PD for mild head injury patients. The chance of developing PD was more for the patients who suffered from loss of consciousness or had severe head injury. Parkinsonian symptoms were associated with neuronal mass loss in substantia niagra with accumulation of a-synuclein in Lewy body and p-tau inclusion in basal ganglia. This reporting patient had severe head injury who also suffered from loss of consciousness.

The most atypical feature of this patient was the short onset of the PD. It will be interesting to follow the patient in long term and if possible to compare the features if similar patients were found in this hospital. The author leaves the opportunity open to make a case series in near future with the similar patients to create a strong hypothesis on the developing PD features immediately after severe head injury.

In conclusion, developing Parkinsonism symptoms immediately after head injury is a rare scenario. Only few cases have been reported in past. It will be interesting to follow future cases to compare and contrast with this case.
